# Decomposed liver has a significantly adverse affect on the development rate of the blowfly *Calliphora vicina*

**DOI:** 10.1007/s00414-012-0697-3

**Published:** 2012-04-26

**Authors:** Cameron S. Richards, Catherine C. Rowlinson, Lue Cuttiford, Rebecca Grimsley, Martin J. R. Hall

**Affiliations:** 1 Department of Entomology, Natural History Museum, Cromwell Rd, London, SW75BD UK; 2 Department of Life Sciences, Imperial College London, Silwood Park, Ascot, SL5 7TG UK

**Keywords:** *Calliphora vicina*, Development rates, Diet, Forensic entomology

## Abstract

The development rate of immature *Calliphora vicina* reared on decomposed liver was significantly slower, by as much as 30 h (55.4 % of total development time) for mid-sized larvae, and 71 h (35.0 %) and 58 h (14.6 %) if using times to the onset of pupariation and eclosion, respectively, than those of immatures that developed on fresh whole pig's liver. Development rates of larvae reared on decomposed liver were also slower than those of larvae reared on minced pig's liver and frozen/thawed pig's liver. These results suggest that any estimate of minimum post-mortem interval may result in an over estimate if the blowflies used were developing on an already decomposed body.

## Introduction

Models describing the development rates of blowfly larvae are the primary tools used by forensic entomologists to estimate a minimum time of death or minimum post-mortem interval (PMI_min_) in cases of suspicious death [4]. There are numerous biotic factors that may affect the development rates of blowfly larvae, and an understanding of these effects is necessary in order to make accurate corrections to a resulting PMI_min_ estimate [2, 8, 12]. One such factor is the effect of diet on the development of immature blowflies and this has been the subject of several recent studies [5–7, 9, 10]. The study by Kaneshrajah and Turner [10] was the first significant contribution to this area and reported differences in the development of *Calliphora vicina* larvae of up to 2 days, after the first 5 days of development at 20 °C, when larvae reared on pig's liver were compared to those reared on pig's brain, heart, kidney or lung tissue. Several notable studies in 2006 looked at a range of diets including the effects of: different organs of one species of animal [6]; the same organ of different animals [5]; different treatments of one organ type, i.e. frozen/thawed vs fresh [7]; and larval densities on a range of different food sources [9]. All these studies have added significant insight into the effects of blowfly larval diet on development, but more work is needed.

It is well documented that blowflies colonise a body soon after death and it is for this reason that they are a good tool to use to estimate PMI_min_ [1, 4, 11]. However, these insects may not always feed on fresh tissue, e.g. in cases where access to a body is delayed, or second and subsequent waves of insect colonisers are used to establish a timeline of events in a case. In these scenarios, the insects will be feeding on decomposed tissue which may have a reduced nutritional value. If this is the case, then it is likely that the development will be retarded, and failure to account for this may have a significant effect on resultant estimates.

This paper investigates the differences in development rates of *C*. *vicina* larvae reared on fresh minced pig's liver and whole pig's liver that was either fresh, thawed from frozen or decomposed.

## Materials and methods

Adult *C*. *vicina* larvae were trapped in the Wildlife Garden of the Natural History Museum and then maintained on sugar and water ad libitum at 21 °C (±2 °C). They were fed 2–4 ml of blood each day for 10 days, after which, they were provided with a ±250-g pork chop as an oviposition medium. Within 1 h, numerous eggs were laid, which hatched within 24 h.

Fresh pig's liver (20 g) was placed into each of ten plastic cups (250 ml). Twenty newly hatched larvae were then added into each cup. These cups were then placed into a LMS (series A1 80 L) incubator set at 23 °C (mean = 23.2 °C; *n* = 673; SD = 0.2). Five larvae, one from each of five cups, were randomly sampled every 6 h for the first 84 h, after which, five larvae were sampled once a day until pupariation. Larvae were killed by immersing them in boiling water for at least 30 s, after which, the length and instar were recorded at each sampling event using a Dinolight Pro2 digital microscope and a Wild M5 light microscope, respectively.

Approximately 5 cm depth of dampened soil was added into each experimental cup, as a pupariation medium, once larvae started showing signs of entering the wandering phase, e.g. emptying of the crop and/or regularly crawling up the sides of the experimental cups. The food and larvae of each cup were placed in a Petri dish, in the experimental cup, on top of the soil. Half of the experimental cups were used to continue sampling larval development, in the manner described above, while the other half of the experimental cups were used to assess pupariation and eclosion. This was achieved by counting the number of larvae, pupae and eclosed adults in one experimental cup at each sampling event. This sampling protocol limits the summary statistic for developmental events data to median data rather than average data.

The average time taken to reach maximum larval size, the median times taken to reach first ecdysis and second ecdysis, the onset of pupariation and the onset of eclosion were calculated. These data and those detailing the development rates of larvae (calculated from length data) were analysed in a Krusal–Wallis (one-way) ANOVA using the statistical software package Statistica 9.

The procedure described above was replicated three times, and the whole experiment was repeated using frozen/thawed pig's liver, fresh minced pig's liver and pig's liver decomposed for 7 days (at 21 ± 2 °C). Liver in the latter treatment was left inside vacuum packaging to prevent drying. Gases generated by the decomposition processes caused the packaging to balloon to its maximum possible size by day 7.

## Results and discussion

There was no significant difference between the three replicates within treatments for all four diet treatments, i.e. fresh whole liver (*F* = 1.32; degrees of freedom (DF) = 30; *p* > 0.13), fresh minced liver (*F* = 1.02; DF = 30; *p* > 0.45), frozen/thawed liver (*F* = 1.77; DF = 30; *p* > 0.12) and decomposed liver (*F* = 1.08; DF = 34; *p* > 0.37). Therefore, all replicates within treatments were combined (*n* = 15 for each sample time per diet treatment) and used to test significant differences between treatments.

### Differences between length data for larvae reared on different diets

**Fig. 1:**
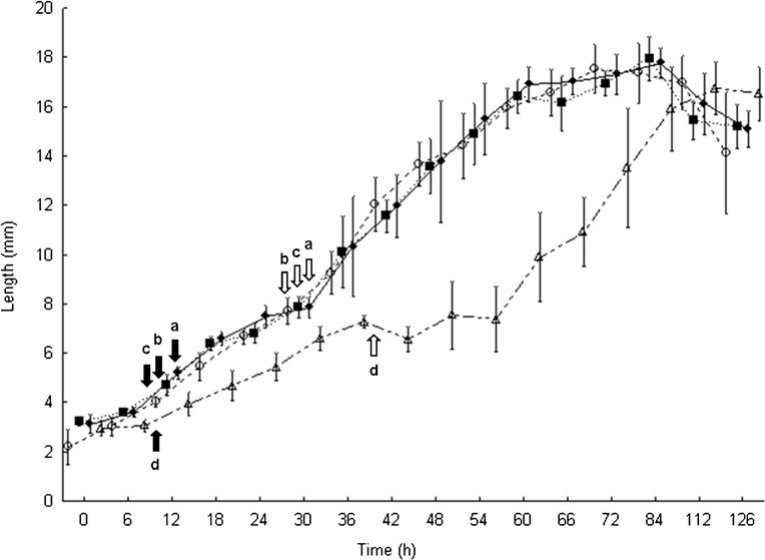
Larval development of *C*. *vicina* on fresh whole (*empty circle*), frozen/thawed (*filled square*), fresh minced (*filled diamond*) and decomposed (for 7 days) (*empty triangle*) pig's liver up to 126 h. *Error bars* represent +1 standard deviation. The *filled* and *empty arrows* on the graph represent the timing of the first and second ecdysis respectively for larvae reared on the (**a**) fresh whole liver, (**b**) frozen/thawed liver, (**c**) fresh minced liver and (**d**) decomposed liver

There was a significant difference in the duration of development for larvae reared on different diets (*F* = 38.1; DF = 45; *p* > 0.00). A Tukey post-hoc test indicated that larval development was significantly slower on decomposed liver than on the other three diets. This significant difference amounted to as much as 30 h of development or 55.6 % of total development time for mid-sized larvae (approx. 7 mm long), but was a consistent difference of 1 day for feeding larvae that were 2 days or older. No significant differences in length data were observed between the other three diets, similar to the findings of Day and Wallman [7] (Fig. 1).

The development rates of larvae (derived from length data) up to second ecdysis were very similar for larvae reared on whole fresh liver (slope = 0.19), fresh minced liver (slope = 0.18) and frozen/thawed liver (slope = 0.17) (Fig. 1) (although those reared on fresh liver were statistically significantly faster (*F*_3,8_ = 66.61; *p* < 0.02) than those reared on frozen/thawed liver, we consider this difference biologically insignificant because they all reached second ecdysis within 5 h of one another (Table 1)). However, larvae, up to second ecdysis, reared on decomposed liver (slope = 0.11) developed statistically and biologically significantly slower (*F*_3,8_ = 66.61; *p* < 0.00) than those reared on the other diets (Fig. 1). On the other hand, the development rates of feeding third instar larvae, larger than 8 mm, reared on all diets were not significantly different (*F*_3,8_ = 2.02; *p* > 0.18) from one another: whole fresh liver (slope = 0.28), fresh minced liver (slope = 0.29), frozen/thawed liver (slope = 0.28) and on decomposed liver (slope = 0.28) (Fig. 1).

**Table 1:** Accumulative median times taken for development of *C*. *vicina* to five developmental events

Developmental event	Development time (h) on different diets			
	Fresh whole	Frozen/thawed	Fresh minced	Decomposed
First ecdysis	13^a^	10^a^	10^a^	10^a^
Second ecdysis	33^a^	28^a^	31^a^	40^b^
Maximum length	70^a^	84^b^	84^b^	112^c^
Pupariation	130^a^	143^b^	140^ab^	201^c^
Eclosion	397^a^	430^b^	422^b^	455^c^

Accumulative median times are the time for 50 % of the experimental population to reach a developmental event; durations to all developmental events exclude egg incubation (*n* = 15; pooled data from three replicates of five samples each). Significant difference within rows (*p* < 0.05) was calculated using one-way ANOVA and denoted by superscript letters accordingly

These data suggest that first, second and early third instar larvae are the larval feeding stages that are adversely affected when reared on decomposed liver, while larvae larger than 8 mm have the ability to develop equally well on decomposed liver as on all other diets tested. A reason for this might be because larger (i.e. oldest) larvae are the most likely to be naturally exposed to decomposed diets and are perhaps best adapted to tolerate them.

### Differences between the duration to developmental events for larvae reared on different diets

Larvae reared on the four different diets reached first ecdysis within 3 h of one another. Larvae reared on fresh whole, frozen/thawed and minced liver reached second ecdysis within 5 h of one another, while those reared on decomposed liver took significantly longer (another 7 h) (*F*_3,8_ = 7.88; *p* < 0.00). Development times to reach the maximum larval length were the same for larvae reared on frozen/thawed and minced liver and differed by only 3 h (2.1 % of total development time) and 8 h (1.9 %) for pupariation and eclosion. On the other hand, larvae developing on whole fresh liver were significantly (14.3 and 7.6 %) quicker than larvae reared on other food sources in reaching the maximum larval length (*F*_3,8_ = 310.33; *p* < 0.00) and eclosion (*F*_3,8_ = 27.22; *p* < 0.00), respectively. Larvae reared on decomposed liver took significantly longer to reach these developmental landmarks, including pupariation (*F*_3,8_ = 161.56; *p* < 0.00), when compared to those reared on the other diets (Table 1). In particular, larvae reared on decomposed liver took 71 h (35.0 %) and 58 h (14.6 %) longer, respectively, to reach pupariation and eclosion when compared to those reared on whole fresh liver (Table 1).

The duration of second instar (*F*_3,8_ = 7.41; *p* < 0.01), feeding third instar (*F*_3,8_ = 53.53; *p* < 0.00) and the wandering phase (*F*_3,8_ = 50.58; *p* < 0.00) were all significantly longer for larvae reared on decomposed liver than the other three diets tested. However, the duration of pupariation (*F*_3,8_ = 24.76; *p* < 0.00) for larvae reared on decomposed liver was significantly shorter than those larvae reared on fresh minced liver (*p* < 0.00) and frozen/thawed liver (*p* < 0.00), but not significantly different from those reared on whole fresh liver (*p* > 0.05) (Table 2).

**Table 2:** Median duration of each developmental stage for *C*. *vicina* (*n* = 15; pooled data from three replicates of five samples each)

Developmental stage	Development time (h) on different diets			
	Fresh whole	Frozen/thawed	Fresh minced	Decomposed
First instar	13^a^	10^a^	10^a^	10^a^
Second instar	20^a^	18^a^	21^a^	30^b^
Feeding third instar	37^a^	56^b^	53^b^	72^c^
Wandering phase	60^a^	59^a^	56^a^	89^b^
Pupariation	267^ab^	287^c^	282^ac^	254^b^

Significant difference within rows (*p* < 0.05) calculated by one-way ANOVA and denoted by superscript letters accordingly

The reason for a lack of effect of decomposed liver on the duration of pupariation may be due to the fact that metamorphosis is not regulated by diet, as the larvae had already finished feeding, but rather by other developmental process. Why the post-feeding larvae were affected after a diet of decomposed liver is presently unknown.

## Conclusions

Decomposed liver had a significantly adverse effect on the development rate of *C*. *vicina*, when compared to the development on fresh whole liver, frozen/thawed liver and minced liver. It is important to note that, in most cases, these differences in rates of development will be unlikely to affect estimates of PMI_min_, because larvae or other stages (i.e. the oldest specimens) used to estimate PMI_min_ will be developing on fresh bodies due to the promptness with which adult parent blowflies can locate a dead body [3]. However, estimates in cases where there was a significant delay in colonisation of the body or those used to establish a timeline of events, e.g. the date of relocation of a badly decomposed body, could result in an over estimate of up to 30 h (55.6 %) if using larvae and up to 71 h (35.0 %) and 58 h (14.6 %) if using times to the onset of pupariation and eclosion, respectively.

It is assumed that the decomposition of pig's liver in this study was primarily due to anaerobic bacteria residing in the liver due to the sealed packaging in which the liver was stored. It is possible that decomposition by aerobic bacteria might have a different effect on the development rates of blowflies, but this was outside the scope of this study. This is something that could be included in future studies, as well as assessing the effects of different stages of decomposition on the development of blowflies and other forensically important insects and determining what factors in decomposed liver are responsible for the observed effects.

Larvae reared on fresh liver required the least amount of time to reach maximum larval length, pupariation and eclosion than those larvae reared on all other diets. Therefore, altering the food substance that blowfly larvae feed on, by either bacterial (represented by the decomposed treatment) or mechanical means (represented by the minced and frozen/thawed treatment) appears to have a negative effect of development.
